# Radiofrequency Echographic Multi-Spectrometry for bone health assessment in prostate cancer patients receiving Androgen deprivation therapy: A prospective comparative observational study

**DOI:** 10.1007/s40520-026-03467-4

**Published:** 2026-08-04

**Authors:** Manuela Montatore, Riccardo Guglielmi, Amanda Isaac, Maria Grazia Capasso, Antonio Corvasce, Federica Masino, Giuseppe Guglielmi

**Affiliations:** 1 Department of Clinical and Experimental Medicine, Foggia University School of Medicine, Viale L. Pinto 1, Foggia, 71121 Italy; 2 Radiology Unit, Mount Sinai Hospital, 600 University Ave, Toronto, ON M5G 1X5 Canada; 3 School of Biomedical Engineering and Imaging Sciences, King’s College London, Strand, London WC2R 2LS, London, UK; 4 Radiology Unit, “Dimiccoli” Hospital, Viale Ippocrate 15, Barletta, 76121 BT Italy; 5 Unit of Urology, L. Bonomo Hospital, ASL BAT, Viale Istria 1, BT 76123 Andria, Italy

**Keywords:** Prostate cancer, PCa, REMS, Ultrasound, Radiofrequency Echographic Multi Spectrometry, ADT

## Abstract

**Background:**

Androgen deprivation therapy (ADT), used in selected patients with prostate cancer (PCa), is associated with accelerated bone loss and increased fragility fracture risk. Radiofrequency Echographic Multi-Spectrometry (REMS) is a non-ionizing ultrasound-based technique for axial bone mineral density (BMD) assessment.

**Aim:**

To evaluate REMS-derived BMD at the lumbar spine and femoral neck in men with PCa receiving ADT and in age- and body mass index-matched healthy controls.

**Methods:**

In this cross-sectional study, 143 Caucasian men were enrolled between October 2023 and October 2024: 83 patients with histologically confirmed PCa receiving ADT for at least 6 months and 60 healthy controls. REMS scans were performed at the lumbar spine and femoral neck. Between-group differences in BMD, T-score, and Z-score were assessed using an unpaired *t*-test.

**Results:**

Compared with controls, patients receiving ADT showed lower REMS-derived BMD at the lumbar spine (0.885 ± 0.08 vs. 0.945 ± 0.11 g/cm²; *p* = 0.007), corresponding to a 6.3% difference. Femoral neck BMD was also lower in the PCa group (0.691 ± 0.08 vs. 0.715 ± 0.10 g/cm²; *p* = 0.01), corresponding to a 3.4% difference.

**Conclusions:**

These findings are consistent with the known skeletal effects of ADT and support the potential role of REMS as a radiation-free tool for identifying reduced axial BMD in men with PCa undergoing ADT. Longitudinal monitoring assessments are ongoing to clarify the temporal relationship between ADT exposure and progressive bone loss.

**Supplementary Information:**

The online version contains supplementary material available at https://doi.org/10.1007/s40520-026-03467-4.

## Introduction

Prostate cancer (PCa) is one of the most common malignancies in older men and represents a major health burden in ageing populations. Androgen deprivation therapy (ADT) remains a cornerstone treatment in selected patients with high-risk, recurrent, or advanced disease. However, suppression of circulating androgens disrupts bone remodeling and is associated with accelerated bone mineral density (BMD) loss, secondary osteoporosis, and increased fragility fracture risk [1].

Currently, a diagnosis of PCa is typically made through a digital rectal examination, prostate-specific antigen (PSA) analysis, prostate biopsies, magnetic resonance imaging (MRI), or routine health screening [2, 3]. Treatment options for localized prostate cancer include active surveillance, ablative radiation, and radical prostatectomy. Men with relapsed or metastatic prostate cancer typically receive ADT, salvage radiation, and chemotherapy. ADT, also known as hormonal therapy, is advised for the treatment of recurrent malignancies, particularly when they have spread beyond the prostate [4, 5]. The therapeutic method is centred on the inhibition of testosterone synthesis and other male hormones, preventing them from fuelling prostate cancer cells. As a result, the significantly reduced levels of male hormones inhibit androgen action on the androgen receptor [6, 7].

Like most of the treatments, ADT comes with potential severe side effects, including toxicity and decreased white and red blood cell counts as well as peripheral neuropathy, erectile dysfunction, incontinence, weariness, hair loss, bone loss, metastases, and finally, the development of resistance against the initial treatment [8, 9]. Furthermore, cancer and cancer therapies increase the risk of osteoporosis by causing bone loss and degeneration of bone microarchitecture, which equally contribute to a decrease in bone strength, leading to increased bone fragility and a higher risk of fractures [10, 11]. This situation not only jeopardizes the patient’s functional status and quality of life but can also have a serious impact on survival [12–14].

Currently, several imaging techniques are available for the evaluation of bone health aiding in the diagnosis of osteoporosis, which include [15–17]: the dual-energy X-ray absorptiometry (DXA), a X-ray based technique evaluating the BMD at reference anatomical site (proximal femur and lumbar spine); the quantitative computed tomography (QCT), another X-ray based technique that enables true volumetric measurements of BMD at lumbar spine and proximal femur, independently of body size; the quantitative ultrasonography (QUS), a ultrasound-based technology that measures the transmission speed and attenuation of waves at peripheral sites including heel, patella, and phalanges; and the Radiofrequency Echographic Multi-Spectrometry (REMS) an ultrasound-based technique for osteoporosis evaluation at reference anatomical sites through the analysis of the raw, unfiltered ultrasound signals acquired during the scans.

Among these, REMS represents an innovative, radiation-free, and portable technology for monitoring bone mineral density (BMD) and bone quality (via Fragility Score, FS) at the lumbar spine and femoral neck. FS is a dimensionless parameter, ranging from 0 (maximum similarity to normal condition) to 100 (maximum similarity to fragile condition), that evaluates skeletal fragility independently of BMD [18, 19]. Scientific evidence shows a higher ability of FS in the prediction of skeletal fragility compared to REMS- and DXA T-score [20]. Thanks to its radiation-free nature, high applicability, and portability, REMS is particularly advantageous in clinical settings where DXA is limited. Its portability, in fact, allows for bedside, emergency, home, and low-resource assessments, facilitating osteoporosis diagnosis in frail patients without the logistical challenges of DXA [21]. Additionally, its non-ionizing nature makes it suitable for vulnerable populations, such as pregnant women and children, enabling more frequent monitoring [22].

This study aimed to assess REMS-derived BMD at the lumbar spine and femoral neck in men with prostate cancer receiving ADT and to compare these values with those of age- and BMI-matched healthy controls.

## Materials and methods

This cross-sectional study was conducted at the Department of Radiology, Dimiccoli Hospital, Barletta, Italy. Patient recruitment started in October 2023 and ended in October 2024. The study was performed in accordance with the principles of the Declaration of Helsinki and was approved by the Institutional Review Board (approval ID: 2258/2011). The study is reported in accordance with the STROBE statement for cross-sectional studies. All participants provided written informed consent before enrolment. All patients in the prostate cancer group were receiving ADT at the time of REMS assessment and had been on treatment for at least 6 months. Detailed information on ADT duration beyond this minimum exposure threshold and on specific ADT regimens was not uniformly available for all patients; therefore, these variables were not included in the comparative analysis.

The prostate cancer group included Caucasian men aged 60–80 years with histologically confirmed PCa who had been receiving ADT for at least 6 months. Exclusion criteria were: a previous diagnosis of secondary osteoporosis or metabolic bone disease, contraindications or technical limitations to ultrasound imaging, and previous or ongoing treatments known to substantially influence bone metabolism, including bisphosphonates, denosumab, or long-term corticosteroids.

The control group consisted of age- and BMI-matched Caucasian healthy volunteers with no known history of bone disorders. Matching was performed according to age and BMI.

### Measurements

REMS scans were performed using an EchoStation device (Echolight S.p.a., Lecce, Italy) equipped with a convex transducer operating at a nominal frequency of 3.5 MHz, following the manufacturer’s instructions. The acquisitions were performed according to procedures described in previously published works [23, 24]. For lumbar spine examinations, the echographic transducer was positioned transabdominally under the sternum to initially visualize the L1 vertebra. Then, the probe is moved down to the L4 vertebra, following the audible instructions from the EchoStudio software (Echolight S.p.a., Lecce, Italy) for a total of 80 s examination. Femoral neck scans were performed by positioning the echographic transducer parallel to the head-neck axis of the femur to visualize the femoral head, neck, and trochanter. Once the target interfaces were identified, the probe was held for 40 s according to the indications provided by the device.

All the REMS acquisitions were performed by operators who had received the specific training program according to the guidelines of the device manufacturer and had at least 3 months of previous experience in REMS acquisitions.

### Statistical analysis

Continuous variables were reported as mean ± standard deviation (SD). Normality was assessed using the Shapiro–Wilk test. Between-group comparisons were performed using an unpaired Student’s *t*-test or Welch’s *t*-test. Mean differences were calculated for REMS-derived BMD, T-score, and Z-score. A two-sided *p*-value < 0.05 was considered statistically significant. Statistical analyses were performed using R software [25].

## Results

143 male participants, including 83 patients with histologically confirmed PCa who had been undergoing ADT for at least 6 months (mean age ± SD: 74.2 ± 7.0 years) and 60 healthy controls (73.5 ± 7.3 years), were enrolled in the study. Table 1 shows demographic, anthropometric, and densitometric data of both groups.

**Table 1:** Baseline Demographic and Densitometric Characteristics of the Study Population

Group	Measurement site	Age (mean ± SD; years)	BMI (mean ± SD; kg/m^2^)	BMD (mean ± SD; g/cm^2^)	T-score (mean ± SD)	Z-score (mean ± SD)
Prostate Cancer (*n* = 83)	Lumbar Spine	74.2 ± 7.0	25.3 ± 2.9	0.885 ± 0.08	-1.9 ± 0.8	-0.7 ± 0.7
	Femoral Neck			0.691 ± 0.08	-1.8 ± 0.7	-0.7 ± 0.6
Healthy Control (*n* = 60)	Lumbar Spine	73.5 ± 7.3	25.3 ± 3.1	0.945 ± 0.11	-1.3 ± 1.0	-0.2 ± 0.9
	Femoral Neck			0.715 ± 0.1	-1.5 ± 0.7	-0.2 ± 0.9

For the lumbar spine acquisitions, the BMD in the PCa group was 0.885 ± 0.08 g/cm², while the BMD in the healthy control group was 0.945 ± 0.11 g/cm^2^. The percentage difference in BMD between the healthy control and the PCa group was 6.3%. An unpaired *t*-test indicated a statistically significant (*p* < 0.05) bone loss at the lumbar spine in patients receiving ADT (Table 2).

**Table 2:** Differences in BMD, T-score, and Z-score values at the lumbar spine between PCa and healthy control groups. Statistical test: unpaired *t*-test

Parameters	Mean difference	*p*-value
BMD	0.06	0.007
T-score	0.56	0.006
Z-score	0.54	0.005

For femoral neck acquisitions, the PCa group showed lower BMD values than controls (0.691 ± 0.08 vs. 0.715 ± 0.10 g/cm²), corresponding to a 3.4% difference. The corresponding differences in T-score and Z-score are reported in Table 3.

**Table 3:** Differences in BMD, T-score, and Z-score values at the femoral neck between PCa and healthy control groups. Statistical test: unpaired *t*-test

Parameter	Mean difference	*p*-value
BMD	0.024	0.01
T-score	0.49	0.01
Z-score	0.45	0.02

## Discussion

In this cross-sectional study, men with prostate cancer receiving ADT showed lower REMS-derived BMD values at both the lumbar spine and femoral neck compared with age- and BMI-matched healthy controls. The difference was more pronounced at the lumbar spine, where BMD was 6.3% lower in the ADT group, while femoral neck BMD showed a 3.4% reduction. These findings are consistent with the recognized skeletal consequences of ADT and support the importance of systematic bone health assessment in men undergoing hormonal treatment for prostate cancer [26–28].

ADT-induced hypogonadism is known to disrupt bone remodelling, accelerate bone resorption, and increase the risk of secondary osteoporosis and fragility fractures. Previous studies have shown that bone loss may occur early after treatment initiation, particularly during the first year of androgen suppression, with reported BMD reductions ranging from approximately 2.3% to 5.6% across skeletal sites [29–31]. In this context, the lower BMD values observed in our ADT cohort are biologically plausible and clinically relevant, especially considering the older age of the study population and the established relationship between reduced BMD and fracture risk [32–35].

The lumbar spine appeared to be the anatomical site most affected in our cohort. This finding may reflect the higher metabolic activity of trabecular bone, which is particularly sensitive to endocrine changes and accelerated bone turnover. Although the femoral neck also showed lower BMD values in the ADT group, the magnitude of the difference was smaller. This site-specific pattern should be interpreted cautiously, because the study did not include baseline pre-ADT measurements and therefore cannot determine the individual rate of bone loss over time. Nevertheless, the results reinforce the need for early skeletal assessment when ADT is initiated or continued, in order to identify patients who may benefit from preventive or therapeutic interventions.

REMS may have a useful role in this clinical setting because it provides a non-ionizing and portable method for BMD assessment at clinically relevant axial sites, including the lumbar spine and femoral neck [36–38]. Unlike peripheral quantitative ultrasound techniques, REMS evaluates reference skeletal sites commonly used in osteoporosis assessment, while avoiding ionizing radiation exposure. This feature may be advantageous in older or frail patients, in those requiring repeated monitoring, and in settings where access to DXA is limited. The present findings suggest that REMS may represent a complementary radiation-free tool for bone-health assessment in selected high-risk populations [39, 40].

The clinical relevance of these findings is strengthened by the increasing attention to cancer treatment-induced bone loss. Men with prostate cancer receiving ADT represent a population in whom fracture prevention should be integrated into routine care, including baseline skeletal assessment, evaluation of clinical risk factors, adequate calcium and vitamin D intake, lifestyle measures, and pharmacological treatment when indicated. In this framework, REMS could contribute to earlier identification of patients with reduced BMD and support longitudinal monitoring, particularly when repeated assessments are needed. Similar applications of REMS have recently been explored in other oncology-related endocrine treatment settings, including women with breast cancer receiving aromatase inhibitors, suggesting a broader potential role for this technique in cancer treatment-induced bone loss.

From a clinical perspective, REMS may represent a useful tool to integrate bone-health assessment into the routine management of men receiving ADT. Its radiation-free nature, portability, and ability to assess axial skeletal sites may facilitate baseline evaluation before or shortly after ADT initiation and support longitudinal monitoring during treatment. This may be particularly relevant in settings where DXA availability is limited, where repeated assessments are needed, or where a rapid, non-ionizing approach could improve access to skeletal-risk evaluation. In this context, REMS should be considered as a complementary technique that may help identify patients who require closer bone-health surveillance and multidisciplinary management.

This study has several limitations. First, it was conducted at a single centre and included only Caucasian men, which may limit the generalizability of the findings. In addition, detailed information on ADT duration beyond the minimum inclusion criterion of 6 months, specific ADT regimens, vitamin D supplementation, calcium intake, smoking status, alcohol consumption, previous fragility fractures, and physical activity was not uniformly available. These factors may influence bone health and could have contributed to residual confounding. Therefore, although the two groups were matched for age and BMI, unmeasured clinical and lifestyle-related variables may have affected the observed differences in REMS-derived BMD. Second, the absence of pre-ADT baseline BMD measurements prevents assessment of individual bone loss after treatment initiation and does not allow causal inference. Nevertheless, the groups were matched for age and BMI, which are key factors influencing BMD, thereby enhancing the validity of the comparison. Third, longitudinal monitoring assessments are ongoing in order to clarify the temporal relationship between ADT exposure and progressive bone loss.

Longitudinal monitoring of this cohort is ongoing. Planned follow-up assessments are intended to evaluate changes in REMS-derived BMD over time, with particular attention to the temporal relationship between ADT exposure and progressive bone loss. Future analyses will also aim to explore clinically relevant outcomes, including incident fragility fractures, treatment-related skeletal deterioration, and the potential role of REMS-derived parameters in fracture-risk stratification.

## Conclusion

In conclusion, men with prostate cancer receiving ADT showed lower REMS-derived BMD at the lumbar spine and femoral neck compared with age- and BMI-matched healthy controls, with the most pronounced difference observed at the lumbar spine. These findings are consistent with the known skeletal effects of ADT and support the potential role of REMS as a radiation-free and accessible tool for bone-health assessment in this high-risk population. However, the cross-sectional design, lack of direct DXA comparison, absence of pre-ADT baseline measurements, and limited availability of treatment- and lifestyle-related variables restrict causal and prognostic interpretation. Further longitudinal studies are warranted to clarify whether REMS-derived parameters may support monitoring and fracture-risk stratification in men undergoing ADT.

## Supplementary Information

Below is the link to the electronic supplementary material.

Supplementary Material 1

## Data Availability

The datasets generated and/or analysed during the current study are not publicly available due to privacy and ethical restrictions, but may be made available from the corresponding author upon reasonable request and under a dedicated data-sharing agreement.

## Declarations

### AI-statement

AI-assisted language editing was used to improve readability and grammar. All scientific content was reviewed and approved by the authors.
