# Supplementary material for: Radiofrequency Echographic Multi-Spectrometry for bone health assessment in prostate cancer patients receiving Androgen deprivation therapy: A prospective comparative observational study
Source: Aging Clin Exp Res. 2026 Aug 4;38(1):224. doi: 10.1007/s40520-026-03467-4 (PMC13736453; doi:10.1007/s40520-026-03467-4)
Supplement: Supplementary file 1 [file 40520_2026_3467_MOESM1_ESM.docx]

**STROBE Statement—Checklist of Items for Observational Studies**

Prepared for ACER submission | ADT-REMS manuscript

Manuscript: Radiofrequency Echographic Multi-Spectrometry for Bone Health Assessment in Prostate Cancer Patients Receiving Androgen Deprivation Therapy: A Cross-Sectional Study

Journal: Aging Clinical and Experimental Research | Article type: Research

| **Section** | **Item no.** | **STROBE recommendation** | **Reported in manuscript** |
| --- | --- | --- | --- |
| Title and abstract | **1(a)** | Indicate the study design with a commonly used term in the title or the abstract. | Title page; Abstract—Methods section. |
| Title and abstract | **1(b)** | Provide in the abstract an informative and balanced summary of what was done and what was found. | Structured Abstract: Background, Aim, Methods, Results, Conclusions. |
| Introduction: Background/rationale | **2** | Explain the scientific background and rationale for the investigation being reported. | Introduction. |
| Introduction: Objectives | **3** | State specific objectives, including any prespecified hypotheses. | End of Introduction. |
| Methods: Study design | **4** | Present key elements of study design early in the paper. | Abstract—Methods; Materials and Methods. |
| Methods: Setting | **5** | Describe the setting, locations, and relevant dates, including periods of recruitment, exposure, follow-up, and data collection. | Materials and Methods: Department of Radiology, Dimiccoli Hospital, Barletta, Italy; recruitment October 2023 to October 2024. |
| Methods: Participants | **6(a)** | Cross-sectional study: give the eligibility criteria, and the sources and methods of selection of participants. | Materials and Methods: inclusion and exclusion criteria for prostate cancer patients and healthy controls. |
| Methods: Participants | **6(b)** | For matched studies, give matching criteria and number of exposed and unexposed participants. | Abstract—Methods; Materials and Methods; Results; Table 1. Age- and BMI-matched groups: 83 PCa patients receiving ADT and 60 healthy controls. |
| Methods: Variables | **7** | Clearly define all outcomes, exposures, predictors, potential confounders, and effect modifiers. Give diagnostic criteria, if applicable. | Materials and Methods and Measurements. Main variables: ADT exposure; REMS-derived BMD, T-score and Z-score at lumbar spine and femoral neck; age and BMI matching. |
| Methods: Data sources/measurement | **8** | For each variable of interest, give sources of data and details of methods of assessment. Describe comparability of assessment methods if there is more than one group. | Measurements. REMS device, acquisition protocol and operator training are described; same assessment method used in both groups. |
| Methods: Bias | **9** | Describe any efforts to address potential sources of bias. | Materials and Methods: age- and BMI-matching; Discussion: limitations and residual confounding. |
| Methods: Study size | **10** | Explain how the study size was arrived at. | Abstract—Methods and Results report the included sample size: 143 participants, including 83 ADT-exposed patients and 60 controls. Formal sample-size calculation not reported. |
| Methods: Quantitative variables | **11** | Explain how quantitative variables were handled in the analyses. If applicable, describe which groupings were chosen and why. | Statistical analysis. BMD, T-score, Z-score, age and BMI are reported as continuous variables; group comparisons are reported in Tables 1–3. |
| Methods: Statistical methods | **12(a)** | Describe all statistical methods, including those used to control for confounding. | Statistical analysis. Normality assessment, unpaired Student t-test or Welch t-test, mean differences, two-sided p-value threshold and R software are reported. |
| Methods: Statistical methods | **12(b)** | Describe any methods used to examine subgroups and interactions. | Not applicable/not performed. |
| Methods: Statistical methods | **12(c)** | Explain how missing data were addressed. | Not explicitly reported; no missing-data analysis presented in the submitted manuscript. |
| Methods: Statistical methods | **12(d)** | If applicable, describe analytical methods taking account of sampling strategy. | Not applicable. |
| Methods: Statistical methods | **12(e)** | Describe any sensitivity analyses. | Not applicable/not performed. |
| Results: Participants | **13(a)** | Report numbers of individuals at each stage of study: potentially eligible, examined for eligibility, confirmed eligible, included, completing follow-up, and analysed. | Results. Included and analysed participants are reported: 83 PCa patients receiving ADT and 60 healthy controls. Screening/exclusion flow not reported. |
| Results: Participants | **13(b)** | Give reasons for non-participation at each stage. | Not reported/not applicable to the presented dataset. |
| Results: Participants | **13(c)** | Consider use of a flow diagram. | Not included; optional for this submission. |
| Results: Descriptive data | **14(a)** | Give characteristics of study participants and information on exposures and potential confounders. | Results and Table 1. |
| Results: Descriptive data | **14(b)** | Indicate number of participants with missing data for each variable of interest. | Not explicitly reported. |
| Results: Descriptive data | **14(c)** | Cohort studies: summarise follow-up time. | Not applicable; this is a cross-sectional analysis and no longitudinal follow-up outcomes are reported. |
| Results: Outcome data | **15** | Report numbers of outcome events or summary measures. | Results and Tables 1–3. REMS-derived BMD, T-score and Z-score are reported. |
| Results: Main results | **16(a)** | Give unadjusted estimates and, if applicable, confounder-adjusted estimates and their precision. Make clear which confounders were adjusted for and why. | Results and Tables 2–3. Unadjusted group differences and p-values are reported. |
| Results: Main results | **16(b)** | Report category boundaries when continuous variables were categorized. | Not applicable; quantitative variables are presented as continuous measures. |
| Results: Main results | **16(c)** | If relevant, consider translating estimates of relative risk into absolute risk for a meaningful time period. | Not applicable; fracture outcomes and time-to-event risk estimates are not reported. |
| Results: Other analyses | **17** | Report other analyses done, such as analyses of subgroups and interactions, and sensitivity analyses. | Not applicable/not performed in the current manuscript. |
| Discussion: Key results | **18** | Summarise key results with reference to study objectives. | Discussion and Conclusion. |
| Discussion: Limitations | **19** | Discuss limitations of the study, taking into account sources of potential bias or imprecision. Discuss both direction and magnitude of any potential bias. | Discussion: limitations paragraph. |
| Discussion: Interpretation | **20** | Give a cautious overall interpretation of results considering objectives, limitations, multiplicity of analyses, results from similar studies, and other relevant evidence. | Discussion and Conclusion. |
| Discussion: Generalisability | **21** | Discuss the generalisability/external validity of the study results. | Discussion: limitations paragraph, including single-centre design and Caucasian male population. |
| Other information: Funding | **22** | Give the source of funding and the role of the funders for the present study and, if applicable, for the original study on which the present article is based. | Funding statement. |
